# Sonographic Evaluation of the Gallbladder in Adult Patients With Type 2 Diabetes Mellitus

**DOI:** 10.7759/cureus.23920

**Published:** 2022-04-07

**Authors:** Teddy A Ikhuoriah, Olubunmi Olatunji, Biodun Adeyinka, David Oboh

**Affiliations:** 1 Department of Radiology, New York Institute of Technology College of Osteopathic Medicine, Old Westbury, USA; 2 Department of Radiology, National Hospital Abuja, Abuja, NGA; 3 Department of Radiology, University College Hospital, Ibadan, NGA

**Keywords:** gallbladder wall thickness, gallstone, type 2 diabetes mellitus, ultrasonography, gallbladder

## Abstract

Introduction and aim: Diabetes mellitus (DM) is one of the most common non-communicable diseases worldwide. Diabetics with autonomic neuropathy tend to have larger gallbladder (GB) with poor contraction after fatty meals predisposing them to gallstones and cholecystitis. This may be prevented and treated if detected early using ultrasound. This study sonographically evaluated the GB in adults with type 2 diabetes and compared the findings with a non-diabetic age and sex-matched control group.

Methods: In this case-control study, 120 patients with type 2 diabetes and 120 non-diabetic controls between the ages of 18 and 80 years at National Hospital Abuja had their GB evaluated after eight hours of overnight fast using B-mode ultrasound. The data were analyzed using IBM SPSS version 20.0 (Armonk, NY: IBM Corp.) and presented in tables.

Result: There were 60 males and 60 females with mean ages of 53.3 and 52 years for the cases and controls, respectively. The average fasting gallbladder volume (FGBV) in diabetics (34.51 + 3.16cm^3^) was higher than that of controls (27.17 + 1.25cm^3^). Eleven (9.2%) diabetics had gallstone (GS), while none was detected in controls. The GB wall thickness was significantly higher in diabetics than in the controls (0.28 ± 0.06 cm vs 0.25 ± 0.04 cm).

Conclusion: A significant proportion of type 2 diabetics had higher FGBV, GB wall thickness, and presence of gallstone compared to the non-diabetic controls. B-mode ultrasound is a very important non-invasive and accurate tool for detecting these changes early.

## Introduction

Diabetes mellitus (DM) is an irreversible syndrome of chronic hyperglycemia due to relative insulin deficiency, resistance, or both [1]. Diabetes is broadly classified into type 1 and type 2. Type 2 is the most common type accounting for at least 90% of cases [1,2]. The current prevalence of DM in Nigeria is estimated to be about 8-10% [3]. In addition to the other complications seen in type 2 DM, they also suffer from autonomic neuropathy, which affects the motility and function of the digestive system including the gallbladder (GB) whose functions are regulated by the autonomic nervous system [1,3,4]. Reduced motility of the GB causes GB hypotonia, the stasis of bile which provides a nidus for stone formation and infection [4,5]. Studies in literature have reported a high prevalence of GB disorder in diabetics compared with the general population, a development which has been hypothesized to result from impairment of GB contractility complicating diabetic vagal neuropathy [4-7]. Real-time ultrasonography is the dominant screening method for GB evaluation and the aim of this study was to sonographically evaluate the GB in patients with type 2 DM and compare the findings with a non-diabetic control group (age and sex matched) [8]. Dodds et al. in 1985 calculated GB volume using a simple ellipsoid method using the following formula: V= π/6 (L x B x H), where L is length, W is the width, and H is the height or anteroposterior (AP) dimensions of the GB; the constant π/6 has a value of 0.523, where π is a constant (22/7) [9]. Chapman et al. in 1998 also compared GB volume in diabetics and control groups using ultrasonography [10], which further corroborated the study by Dodds et al. [9].

## Materials and methods

This was a case-control random study carried out in the Department of Radiology, National Hospital Abuja, Nigeria, where the gallbladders of 120 type 2 diabetic patients and 120 gender and age-matched controls were evaluated using B-mode gray scale ultrasound. To be eligible for this study, cases had to be aged between 18 and 80 years with a confirmed diagnosis of type 2 DM (fasting plasma glucose of >7.0 mmol/L {126 mg/dL} or two hours postprandial glucose equal to or greater than 11.1 mmol/L {200 mg/dL}) and attending the endocrinology clinic or general outpatient department of the hospital. For the control group, the inclusion criterion was consenting healthy non-diabetic age and sex-matched staff of National Hospital Abuja who had fasting plasma glucose concentration values of less than 7.0 mmol/L (126 mg/dL) that were referred to the department for routine abdominal ultrasound as well as other healthy patient relative volunteers. Cases were excluded from the study if they were diabetics on drugs capable of interfering with autonomic function or inhibit cholecystokinin release (alpha methyldopa, atropine, non-steroidal anti-inflammatory drugs, bethanechol, cholestyramine, calcitonin, somatostatin, and octreotide). Patients who had known endocrine-related diseases such as rheumatoid arthritis, systemic sclerosis, systemic lupus erythematosus, and autoimmune thyroiditis that may alter autonomic function were also excluded from the study. Patients who declined consent and pregnant diabetic patients were also excluded. For the control group, subjects less than 18 years of age, patients with fasting blood sugar greater than 126 mg/dl, patients with a recent medical history of jaundice as well as subjects with extreme truncal obesity which made the sonographic demonstration of the GB sub-optimal were excluded. Cases and controls who have had previous hepatobiliary surgery, as well as diabetics and controls with history of hemoglobinopathy, were also excluded from the study. Written informed consent was obtained and details of the age, gender, duration of diabetes mellitus (calculated from time of diagnosis in the hospital to time of ultrasonography), current and past medications, parity (for the female subject), height (in meters), and weight (in kilograms) of the patients were measured and recorded. The body mass index (BMI) was calculated using the formula: weight/height^2^ while the body surface area (BSA) was calculated using the formula: (weight) 0.425 x (height) 0.725 x 0.20247 (Du Bois formula). Diabetics and controls were examined in the mornings after an overnight fast of at least eight hours. Fasting blood sugar (FBS) levels were measured using calibrated test strips of an Accu-check Active glucometer (Indianapolis, IN: Roche Diagnostics).

The ultrasound examinations were performed using a 3.5-5.0 MHz curvilinear array probe of a Philips HD III XE (Amsterdam, the Netherland: Philips) ultrasound machine manufactured in 2012. Each subject was placed on the examination couch in the supine position. The abdomen was exposed with the subject’s hands placed under the head. For optimal visualization of the entire GB, some subjects were scanned in the left lateral decubitus or erect position to avoid missing gallstone (GS) in only supine scans. The GB location was visualized by following the reflective main lobar fissure from the right portal vein to the GB fossa. The image was frozen on the screen and using the electronic calipers; the maximum length (L) of the GB was measured across its central longitudinal axis and recorded [11,12]. The GB was also scanned transversely from the fundus to the neck leading to the cystic duct by orienting the ultrasound probe 90˚ to the long axis of the GB. The maximum diameter of the GB in this plane was obtained after the image was frozen. Measurement from the inner wall of the GB on one side to the inner wall on the opposite side, the maximum width (w) and height (H), or AP dimension were taken and recorded in centimeters [11,12]. The measurements were taken thrice, and an average was calculated for each, this was done to reduce intra-observer error and used to compute the fasting gallbladder volume (FGBV) in cubic centimeters, using the following common ellipsoid formula: V =π/6(L X W X H), where the constant π/6 = 0.52387 [11-13]. GB wall thickness was measured in millimeters (mm) from the inner to the outer wall of the GB on the transverse axis. Other findings such as gallstones and biliary sludge were documented. Data collected were entered and analyzed using IBM SPSS Statistics for Windows, version 20.0 (Armonk, NY: IBM Corp.). The correlation was done using chi-square, t-test, and Fisher’s exact test as applicable. P-value was considered significant when <0.05.

## Results

Two hundred and forty subjects comprising 120 type 2 diabetics and 120 controls participated in the study. The mean age of the diabetics was 53.3 ± 11.4 years, while that of the controls was 52.0 ± 11.8 years. There were no statistical differences in their ages (p = 0.395) (Table 1). The diabetics had higher fasting blood sugar than the controls; however, this was not significant (p = 0.232) (Table 1). The type 2 diabetics also had a significantly higher body weight than the controls (77.9 ± 13.3 kg vs 73.8 ± 13.5 kg; p = 0.019). The BMI of the cases was also higher than the controls but was not significant (p = 0.232) (Table 1).

**Table 1:** Age and anthropometric parameters of study subjects BSA: body surface area; FBS: fasting blood sugar

Demographics	Diabetics, n = 120		Controls, n = 120		Statistical t-test	p-Value
	Mean ± SD	Range	Mean ± SD	Range		
Age (years)	53.3 ± 11.4	21.0-83.0	52.0 ± 11.8	23-75	0.852	0.395
Weight (kg)	77.9 ± 13.3	57.0-110.0	73.8 ± 13.5	46.0-110.0	2.364	0.019
BMI (kg/m^2^)	27.6 ± 4.6	17.79-40.04	26.9 ± 5.0	16.9-38.3	1.199	0.232
BSA (m^2^)	1.90 ± 0.18	1.61-2.32	1.84 ± 0.19	1.40-2.31	2.726	0.007
FBS (mg/dl)	130.1 ± 49.7	64.8-355.0	92.4 ± 14.6	59-113	3.191	0.232 ​​​​​​​

The gallbladder length was significantly higher in the type 2 diabetics compared to the controls (7.75 ± 1.25 vs 6.64 ± 1.09 cm; p = <0.001), but their anteroposterior (AP) and transverse diameters did not differ significantly (p = 0.484 and 0.431, respectively) (Table 2). The mean fasting gallbladder volume (FGBV) for type 2 diabetics 34.51 ± 3.16 cm^3^, while that for controls 27.17 ± 1.25 cm^3^ which was statistically significant (p = 0.032) (Table 2). A similar finding was also noted concerning the Gallbladder wall thickness when the diabetics and controls were compared (0.28 ± 0.06 cm vs 0.25 ± 0.04 cm; p = <0.001) (Table 2).

**Table 2:** Gallbladder measurements in type 2 diabetics and controls AP: anteroposterior

Gallbladder measurements	Diabetics, n = 120		Controls, n = 120		Statistical t-test	p-Value
	Mean ± SD	Range	Mean ± SD	Range		
AP diameter (cm)	2.48 ± 0.58	1.45-5.80	2.42 ± 0.59	1.25-4.67	0.702	0.484
Length (cm)	7.75 ± 1.25	5.64-15.30	6.64 ± 1.09	2.23-8.95	7.348	<0.001
Transverse diameter (cm)	3.14 ± 0.72	1.96-7.64	3.07 ± 0.74	1.68-4.83	0.789	0.431
Fasting gallbladder volume (cm^3^)	34.51 ± 3.16	8.41-81.40	27.17± 1.25	5.50-79.89	2.160	0.032
Wall thickness (cm)	0.28 ± 0.06	0.14-0.44	0.25 ± 0.04	0.13-0.39	4.537	<0.001

Comparison of demographic, anthropometry, glycemic index, and gallbladder measurements in study subjects based on gender

There was no significant difference in the gallbladder indices between the male and female diabetics. Female diabetics had higher gallbladder length, and anteroposterior and transverse diameters. The fasting gallbladder volumes were higher in female diabetics (31.11 ± 13.17 cm^3^ vs 30.14 ± 12.06 cm^3^), while the wall thickness was marginally higher in male diabetics but was not significant (Table 3). In the controls, the fasting gallbladder volume (FGBV) was significantly higher in females (25.25 ± 11.65 cm^3^ vs 29.09 ± 15.37 cm^3^; p = 0.032) but the males had a significantly thicker gallbladder wall (0.25 ± 0.05 cm vs 0.24 ± 0.03 cm; p = <0.001) (Table 3). The fasting gallbladder volume (FGBV) and gallbladder wall thickness was significantly higher in type 2 diabetics when compared with the controls (30.14 ± 12.06 cm^3^ vs 25.25 ± 11.65cm^3^; p = 0.026 and 0.28 ± 0.06 cm vs 0.25 ± 0.05cm, p = 0.006, respectively) (Table 3).

**Table 3:** Demographic, anthropometry, and GB measurement in diabetics and controls DM: diabetes mellitus; CON: controls; FGBV: fasting gallbladder volume; AP: anteroposterior: FBS: fasting blood sugar; GB: gallbladder

Demographic				Male				Female				Statistical t-test		p-Value	
				Mean ± SD		Range		Mean ± SD		Range					
Age (years)		DM		54.8 ± 11.3		29.0-80.0		51.7 ± 11.3		21.0-75.0		1.458		0.148	
		CON		53.8 ± 11.4		28.0-75.0		50.2 ± 12.0		23-71		1.689		0.094	
Weight (kg)		DM		78.2 ± 14.5		57.0-108.0		77.7 ± 12.1		58.0-110.0		0.209		0.835	
		CON		73.0 ± 11.9		49.9-110.0		74.5 ± 14.9		46.0-104.0		-0.622		0.535	
BMI (kg/m^2^)		DM		26.1 ± 3.8		17.8-34.1		29.1 ± 4.8		19.9-40.0		-3.718		0.001	
		CON		25.4 ± 3.8		20.3-35.9		28.4 ± 5.6		16.9-38.3		-3.390		0.001	
FBS (mg/dl)		DM		123.3 ± 39.0		75.6-219.6		136.9 ± 58.0		64.8-355.0		-1.500		0.136	
		CON		93.8 ± 20.1		59-113.0		91.3 ± 9.3		79.0-109.0		0.345		0.735	
GB indices AP diameter (cm)		DM		2.39 ± 0.50		1.54-3.95		2.57 ± 0.65		1.45-5.80		-1.674		0.097	
		CON		2.36 ± 0.61		1.40-3.60		2.49 ± 0.56		1.25-4.67		0.702		0.484	
GB indices transverse diameter (cm)		DM		3.06 ± 0.56		2.23-4.26		3.22 ± 0.84		1.96-7.64		-1.245		0.215	
		CON		2.89 ± 0.57		2.03-4.65		3.25 ± 0.83		1.68-4.83		0.789		0.431	
FGBV (cm^3^)	DM		30.14±12.06		15.87-9.44		31.11±13.17		8.41-81.40		-0.418		0.677		
	CON		25.25 ± 11.65		9.08-65.33		29.09 ± 15.37		5.50-79.89		2.160		0.032		
Wall thickness (cm)		DM		0.28 ± 0.06		0.14-0.44		0.27 ± 0.06		0.16-0.40		0.336		0.737	
		CON		0.25 ± 0.05		0.13-0.39		0.24 ± 0.03		0.14-0.28		4.537		<0.001	

Comparison of fasting gallbladder volume and gallbladder wall thickness in obese and non-obese subjects

Though FGBV was higher in obese diabetics, there was no significant difference in FGBV between obese and non-obese subjects in each group (Table 3). The gallbladder wall thickness was also higher in obese diabetics but there was no significant difference in gallbladder wall thickness between obese and non-obese subjects in each group (Table 3).

GB wall thickening in type 2 diabetic subjects and controls

Thirty-nine (32.8%) of the type 2 diabetics had a significantly increased GB wall thickening (p <0.001) when compared to the six (5%) controls who had GB wall thickening. There was however no association between thickened GB wall with age groups and gender in diabetics, though male diabetics and diabetics who were >60 years had a higher proportion of GB wall thickening (p = 0.602 and 0.286, respectively). Obese type 2 diabetics had a significantly increased proportion of GB wall thickening (p = 0.010) (Table 4).

**Table 4:** GB wall thickness in type 2 diabetic subjects and controls GB: gallbladder

Demographic		Thickened GB wall	Normal GB wall	Statistical chi-square test	p-Value
Age (years)	<30	2 (66.7)	1 (33.3)	5.009	0.286
	30-39	12 (85.7)	2 (14.3)	5.009	0.286
	40-49	18 (66.7)	9 (33.3)	5.009	0.286
	50-59	22 (75.9)	7 (24.1)	5.009	0.286
	≥60	26 (57.8)	19 (42.2)	5.009	0.286
Sex	Male	39 (65.0)	21 (35.0)	0.272	0.602
	Female	41 (69.5)	18 (30.5)	0.272	0.602
BMI	Obese	58 (76.3)	18 (23.7)	6.577	0.010
	Non-obese	20 (52.6)	18 (47.4)	6.577	0.010

Correlation between fasting gallbladder volume and GB wall thickness with demographic, BMI, duration of illness/treatment, and FBS

There was a weak negative correlation between female gender and fasting gallbladder volume (r = -0.127; p = 0.167) while no correlation was established with age, BMI, duration of illness and therapy in the type 2 diabetic subjects. In the controls, a weak significant correlation was found between age and fasting gallbladder volume (r = 0.210, p = 0.021). A weak negative correlation was established between gallbladder wall thickness with female gender and BMI in the type 2 diabetic subjects. In the controls, age showed a weak positive correlation with gallbladder wall thickness (r = 0.246, p = 0.007).

Prevalence of gallstones and other incidental findings

Eleven (9.2%) of type 2 diabetic subjects had gallstones while none of the controls had (p = 0.001). Fatty liver was found in 18 (15.0) of the diabetics and none in the controls (p < 0.001).. Sludge and liver cyst were observed in one control each. The incidence of gallstone was higher with increasing age, with about four cases of gallstones being recorded in the sixth decade. Eight (10.4%) obese diabetics had gallstones compared to two (5.3%) non-obese diabetics. Also, more female diabetics had gallstones than males (11.7% vs 6.7%). No significant association was however seen between age, obesity, and gender (p =0.836, p=0.493, and p=0.529, respectively). The FGBV in type 2 diabetics with gallstone was higher than in those without gallstone however the difference in mean was not significant (36.99 ± 13.51 cm^3^ vs 30.36 ± 12.12 cm^3^, p = 0.121). The GB wall thickness was significantly higher in type 2 diabetics than in controls (0.32 ± 0.06 vs 0.27 ± 0.06, p = 0.014). There was a significant association between obesity and fatty liver (p = 0.015). There was however no association between age and gender with incidence of fatty liver in the diabetic subjects.

Correlation of gallstone with age, BMI, gallbladder volume, and wall thickness in type 2 diabetics

There was a weak positive correlation between the presence of gallstone and fasting gallbladder volume (r = 0.257, p = 0.005). Similarly, a weak positive correlation was also noted between the presence of gallstone and gallbladder wall thickness (r = 0.211, p = 0.027). No significant correlation was established between the presence of gallstone, age, BMI, and female gender.

## Discussion

Type 2 diabetics had a significantly increased FGBV compared to the controls in this study - a finding similar to the observations by Osawe et al. [14], Singh et al. [15], Saxena et al. [16], Raman et al. [17] Naik [18], Tahnia et al. [19], and Kayacentin et al. [4]. Generally, the mean and range of FGBV observed in this study were similar to those reported in studies done in other parts of Nigeria and higher than those of European and Asian studies suggesting geographical variation in GB dimensions [14-16,19,20]. This may be attributed to genetic and environmental differences (especially diet) [7].

Diabetic males and females had higher FGBV compared to the corresponding gender in the control group in this study; however, the difference in mean was significant in females alone. This was consistent with the observations of Pagliarulo et al. [5], Saxena et al. [16], and Raman et al. [17]. When the FGBV was re-evaluated based on gender, within each group, females had a higher FGBV compared to males but the difference in mean FGBV was observed to be statistically significant in the controls alone. This suggests that gender difference had less impact compared to the disease state itself on the GB dimension in diabetics unlike that observed in the controls.

In this study, the mean FGBV was higher in obese diabetics than in non-obese diabetics; however, the difference in the mean was not significant (p = 0.124). The lack of significance may be attributed to the small proportion of obese diabetics compared to the non-obese diabetics in the study. In the controls, the non-obese had a marginal increase in FGBV which was not significant when compared with the obese control subjects. FGBV has been reported to correlate with some demographic and health indices in normal subjects; however, in this study, very weak positive and insignificant correlations were observed between age, BMI, duration of illness, and fasting blood sugar with FGBV and a weak negative correlation with the female gender. This is somewhat similar to the observations of Guliter et al. [21] and Agarwal et al. [22]. Though Agarwal et al. and Ugbaja et al. observed a positive correlation between age and BMI as well as a weak positive correlation between FGBV and duration of diabetes, respectively [22,23].

This study showed a significant increase in the GB wall thickness of diabetic subjects compared to controls as the GB wall was thickened (>3mm) in 32.8% of diabetics compared to 5% of the controls (p <0.001). As with FGBV, gender differences did not impact the GB wall thickness in the diabetics; however, in the controls, males had a significantly increased GB wall thickness compared to females (35% vs 30.5%, p = 0.602). GB wall thickening was significantly higher in the obese type 2 diabetics than in the controls. This also affirms the increased risk of gallbladder disease (GBD) associated with the co-existence of type 2 diabetics and obesity [6,16].

Weak non-significant correlations were observed between age, gender, and FBS when compared with GB wall thickness in diabetics, while positive significant correlation was noted between age and GB wall thickness in controls. Mohammed et al. found that GB wall thickness had a positive correlation with BMI and lacked a significant correlation with age [24]. These differences may be attributed to the fact that their study included predominantly younger subjects (over two-thirds of their study populace were below forty years). McGahan et al. in another study including children found a positive correlation between GB thickness and age which was similar to the findings in the non-diabetic group of this study [25]. 

Gallstones were the predominant morbidity in this study accounting for 9.2% in diabetics, while none of the controls had GS. The high incidence of GS found in type 2 diabetics in this study is consistent with reports from most of the studies in the literature [5,6,18,26-29]; however, the rate in this study is slightly lower than the rates reported in other studies (13-35%). Similarly, lower burden of GBD was also found in the control group compared to other studies. Environmental and dietary factors may contribute to variations in GBD burden from one geographical location to another [7,14]. Diabetic subjects with GS had a higher FGBV compared to those without GS; however, this was not significant. The lack of significance may in part be due to the smaller number of subjects in the subgroup with GS. This is similar to the observations of Olokoba et al. [7]. The incidence of GS was found to increase with each age decade with a peak in the sixth decade (50-59 years) though there was no significant association with age a finding similar to the studies by Elmehdawi et al. [26] and Al-yasiri et al. [27].

Females were found to have higher incidence of GS than males, a similar finding to studies by Saxena et al. [16], Raman et al. [17], and Elmehdawi et al. [26]. The higher rate in females has been attributed to the effect of estrogen and progesterone which induce increase in cholesterol secretion and reduces secretion of bile salt by the GB, respectively [28]. Obesity is an established risk factor for the development of GS, seemingly due to increased cholesterol synthesis and secretion [6]. This was confirmed in this study, similar to other studies in the literature [27,29,30]. Significant but weak positive correlations were observed between GS with the FGBV and with GB wall thickness just like Raman et al. [17].

Study limitation

It is noteworthy to mention that this study has the following limitation: there was no similar research done within the immediate geographical region in which this research was conducted. This would have allowed for comparison.

## Conclusions

The study showed that fasting gallbladder volume and GB wall thickness were significantly increased in type 2 diabetics when compared to controls. There is a strong association between GB wall thickening and obesity and an increased incidence of gallstones and fatty liver was noted in type 2 diabetics compared to controls. A positive correlation was also seen between the presence of gallstones and FGBV as well as GB wall thickness. Routine abdominal ultrasound should thus be recommended in the evaluation of type 2 diabetics, especially in those who are obese, where gallbladder volume and GB wall thickness would be assessed. More diabetic centers that offer efficient and cost-effective management of diabetic patients should be established. Also, a more elaborate community-based study with larger study population should be carried out to provide a more robust database.
